# Assessment of eruption source parameters using infrasound and plume modelling: a case study from the 2021 eruption of Mt. Etna, Italy

**DOI:** 10.1038/s41598-023-46160-6

**Published:** 2023-11-13

**Authors:** Silvio De Angelis, Luciano Zuccarello, Simona Scollo, Luigi Mereu

**Affiliations:** 1 School of Environmental Sciences, University of Liverpool, 4 Brownlow Street, Liverpool, L69 3GP UK; 2 Present Address: Istituto Nazionale di Geofisica e Vulcanologia, Sezione di Pisa, Via Cesare Battisti, 53, 56125 Pisa, Italy; 3 Istituto Nazionale di Geofisica e Vulcanologia, Sezione di Catania - Osservatorio Etneo, Piazza Roma, 2, 95125 Catania, Italy; 4 Istituto Nazionale di Geofisica e Vulcanologia, Sezione di Bologna, Bologna, Italy; 5 CETEMPS Center of Excellence, University of L’Aquila, L’Aquila, Italy

**Keywords:** Natural hazards, Volcanology

## Abstract

Atmospheric injection of volcanic ash during eruptions is a threat to aviation. Reliable forecast of airborne ash dispersal relies on empirical and numerical models. Key inputs into these models are so-called eruption source parameters such as the rate at which pyroclastic material is ejected from the vent and the maximum height of eruptive columns. Here, we use infrasound data recorded during eruptive activity in June 2021 at Mt. Etna, Italy, to demonstrate its potential for assessment of eruption rates in near-real time. We calculate a time series of flow velocity at the vent using data corrected for topographic scattering, and the effect of vent geometry on the acoustic source radiation. We obtain values of flow velocity of 50–125 m/s during a period of sustained, paroxysmal, activity. We use independent estimates from other ground-based remote sensing data to validate our results. Further, we use the infrasound-derived flow velocities as input into a 1D plume model to estimate the maximum height of the eruption column. Our results suggest that infrasound technology holds promise to assess eruption rates and inform modelling of volcanic plumes. We anticipate that implementation of real-time operational workflows based on infrasound data and plume modelling will impact decision-making and risk mitigation at active volcanoes.

## Introduction

### Challenges in monitoring volcanic plumes and ash transport

Explosive eruptions frequently release significant amounts of fine rock particles and glass fragments, referred to as volcanic ash, into the atmosphere. The emission of volcanic ash, owing to its highly abrasive nature and the low melting point of its glass component, can cause severe damage to aircrafts^1–3^. Nine Volcanic Ash Advisory Centres (VAACs) are deputed to evaluate the presence and extent of volcanic ash in the atmosphere, and to issue ash-cloud warnings to the aviation community around the world^1^. The ability of these operational centres to forecast the movement of ash in the atmosphere, and thus identify at-risk regions, is underpinned by complex numerical models. Such models require a set of input parameters that represent the meteorology of the atmosphere at the time of eruption (e.g., wind speed and direction, vertical temperature profile) and the nature of the eruptive source (e.g., source location, time and duration of eruption, vent geometry, height of the plume, mass eruption rate, particle size distribution, ash density and shape)^4^. Constraining the extensive parametrization of atmospheric ash transport models is challenging, and thus, their outputs are affected by substantial uncertainty. Meteorological parameters are usually well-constrained by data and models with temporal and spatial resolutions of the order of hours and few kilometers, respectively (e.g., the Met Office Unified Model^5^ used by the London VAAC^4^). Conversely, larger uncertainties are linked to estimates of eruption source parameters^6, 7^ (ESP), in particular mass or volume eruption rates (MER or VER, respectively). Commonly, VER is estimated through inversion of so-called 0D eruption plume models (EPMs), that is empirical scaling relationships between VER and the height reached by the plume above the vent. The simplest, and most widely used, family of EPMs takes the general form $H=C{V}^{n}$, where $H$ is the maximum height reached by the plume above the vent, $V$ is VER (measured as ${\mathrm{m}}^{3}$ of dense-rock equivalent per second), $C$ and $n$ are constants^1, 8^. For the purpose of informing volcanic ash transport modelling, 1D integral EPMs have also been increasingly tested owing to their ability to include processes such as the entrainment of atmospheric air into the eruption column, and to account for the effect of wind speed and direction on plume rise^4, 9^. The main challenge in the use of EPMs for assessment of VER is that they require validation, which depends on the availability of independent measurements of both plume heights and MER/VER^1, 10, 11^. Independent column height estimates are available from either satellite and ground-based (e.g., camera, radar, lidar) measurements albeit with limitations posed by data latency, logistics and weather conditions. The majority of independent VER estimates are average measurements of the ratio between the total volume of erupted tephra and the duration of the eruptive event. These data can exhibit significant scatter^4, 12^, which arises from differences in the techniques employed to evaluate the volume of tephra deposits and the duration of eruptions, and factors such as the impact of wind speed and direction on the rise of volcanic plumes. Therefore, the need for new methods to quantify volcanic emissions has emerged as a critical step towards improving our capacity to assess and mitigate hazards from volcanic ash.

### Measurements of volume eruption rate

In recent years, a series of studies have proposed methods to estimate VER/MER from analyses of ground-based radar, thermal infrared (IR), and acoustic data. Gouhier and Donnadieu^13^ and Freret-Lorgeril et al.^14^ used echo power data from Doppler Radar measurements to estimate MER at Stromboli and Mt. Etna (Italy), respectively; Marzano et al.^15^ proposed a workflow to measure MER using microwave weather radar and infrasound array data collected during eruptions at Eyjafjallajökull (Iceland, 2010), Grímsvötn (Iceland, 2011) and Mt. Etna (2013); Ripepe et al.^16^ employed a combination of thermal camera imagery and infrasound array data to evaluate plume exit velocity during the 2010 Eyjafjallajökull eruption; Calvari et al.^17^ calculated the exit velocity of the eruptive jet from thermal IR data during paroxysmal activity at Mt. Etna in 2020–2021. Among these methods, infrasound has emerged as a promising tool to quantify VER/MER owing to the comparatively low costs associated with sensor installation and maintenance, data availability in real-time, and its suitability for automated data processing. A substantial body of research has informed continuous development of methods for inversion of acoustic waveforms aimed at quantifying volcanic emissions. Caplan-Auerbach e al.^18^, Lamb et al.^19^, Ripepe et al.^16^ used scaling laws that link the power radiated by acoustic sources to gas velocity during flow from a volcanic vent^20^. Johnson et al.^21^ and Johnson and Miller^22^ used a monopole source model, that is a compact source that radiates acoustic waves hemispherically, to quantify volcanic emissions at Mt. Erebus (Antarctica) and Sakurajima volcano (Japan), respectively. Kim et al.^23^ introduced a waveform inversion method to calculate VER at Tungurahua volcano (Ecuador) based on the Green’s Function solution to the inhomogeneous Helmoltz wave equation in a half-space; the method, based on the representation of the acoustic pressure wavefield as a combination of monopole and dipole terms, was also applied by De Angelis et al.^24^ at Santiaguito volcano (Guatemala). Progress in numerical modelling of the acoustic wavefield generated by compact volcanic sources^25, 26^ underpinned additional studies focussed on retrieval of VER via full waveform inversion of infrasound signals using 3D numerical Green’s Functions^27–30^. A comprehensive review of these methods and their underlying theoretical frameworks can be found in De Angelis et al.^31^. More recently Hantusch et al.^32^ and Freret-lorgeril et al.^33^ have shown that VER can be calculated from direct integration of the acoustic pressure wavefield after applying corrections for scattering from topography, wavefield directivity (controlled by vent geometry and the acoustic wavenumber) and reflectance at the conduit outlet. Hantusch et al.^32^ accounted for topographic scattering of the acoustic wavefield at Copahue volcano (Argentina) via the insertion loss (IL) parameter in the screen diffraction approximation. Freret-Lorgeril et al.^33^ assumed that topographic scattering at Mt. Etna was negligible as the position of the infrasound array used was in line-of-sight with the erupting vent. Maher et al.^34^ demonstrated that the screen diffraction approximation is only appropriate under specific conditions and suggested that numerical simulations are the best tool to estimate IL; a workflow for calculating maps of IL based on numerical modelling of acoustic wave propagation over volcanic topography was proposed by Lacanna and Ripepe^26^.

Here, we build on this extensive body of research and demonstrate a methodology to obtain independent estimates of VER from the integration of attenuation-corrected infrasound data recorded at Mt. Etna, Italy. We benchmark our results with independent estimates of flow velocity at the vent obtained from analyses of ground-based thermal IR imagery. Finally, we show how VER can be used as a direct input into 1D plume models for rapid assessment of maximum column height, thus, providing key monitoring information when other observations may not be available. The results of plume modelling are further validated using ground-based X-band radar data and satellite imagery gathered during eruption.

### Eruptive activity at Mt. Etna: June 2021

**Figure 1:**
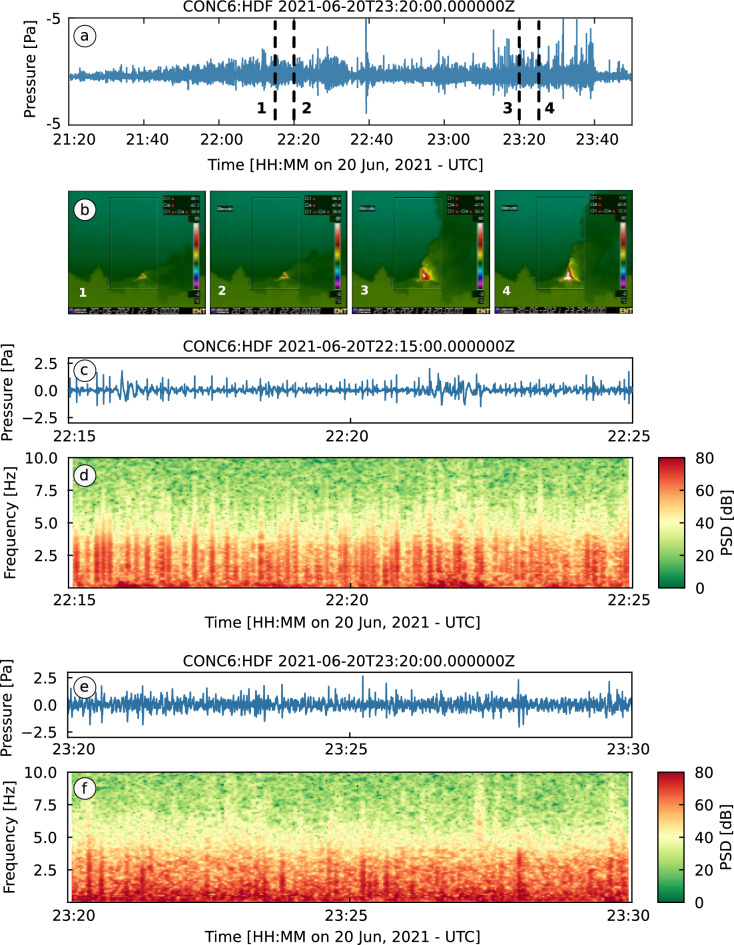
Infrasound and thermal IR data recorded during the paroxysmal activity at Etna on 20 June, 2021. (**a**) Pressure waveform recorded through a paroxysmal event on 20 June, 2021. Infrasound data are recorded by the central sensor of a temporary 6-element infrasound array at approximately 6 km from the active vent (see Fig. 2a–c); (**b**) Four images recorded by a thermal IR camera of the Istituto Nazionale di Geofisica e Vulcanologia, Osservatorio Etneo (INGV-OE), located approximately 15 km South of the Etna summit craters (see Fig. 2a). The images correspond to times 1–4 marked by dashed lines in (**a**); (**c**) Zoomed waveform showing 15 min of acoustic data during Strombolian activity; (**d**) Spectrogram of the signal in (**c**). The spectrogram is calculated as the Power Spectral Density of the pressure time series over a sliding window of 10.24 s with 50% overlap; (**e**) Zoomed waveform showing 10 min of acoustic data during lava fountaining activity; (**f**) Spectrogram of the signal in e) calculated with the same parameters as in (**d**).

Mt. Etna, Italy, is one of the most active volcanoes in the world. One of its distinctive features is the frequent occurrence of so-called paroxysms, that is episodes of intense explosive activity lasting from tens of minutes to many hours^35^. Paroxysms typically occur in clusters within eruptive periods that can last from few weeks to months^36^. Between December 2020 and February 2022 Mt. Etna produced 66 paroxysms^17^. In this study, we focus on an episode on 20 June, 2021, part of a sequence of paroxysms that occurred, with striking regularity, during the second half of June 2021. These events had durations of up to few hours and all followed the characteristic pattern of paroxysmal activity at Mt. Etna. Initial rapid-fire Strombolian explosions evolved into nearly continuous lava fountaining, feeding lava flows and volcanic plumes with heights of up to several kilometers above the vent^37^. Infrasound and thermal IR data recorded during the paroxysmal activity on 20 June are shown in Fig. 1a–f. Figure 1c, d illustrate early Strombolian activity, consisting of discrete explosions clearly distinguishable in both the waveform (Fig. 1c) and spectrogram (Fig. 1d). During the later stages of the paroxysm, the acoustic signal evolves into nearly continuous tremor (Fig. 1e) and individual explosions are only occasionally discerned (Fig. 1f). Figure 1b shows thermal IR images tracking the change from discrete explosions to lava fountains feeding a sustained volcanic plume.

## Data

### Infrasound array

**Figure 2:**
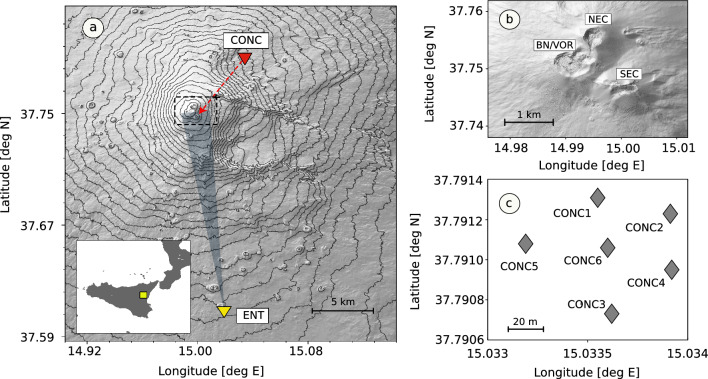
Map of Mt. Etna (digital elevation model data source: https://tinitaly.pi.ingv.it/) and location of infrasound and thermal IR sensors used in this study. (**a**) Map showing the locations of temporary infrasound array CONC (red triangle) and thermal IR ENT camera (yellow triangle). CONC was installed at the site Monte Conca, approximately 6 km from the active vent; ENT operated by the Istituto Nazionale di Geofisica e Vulcanologia, Osservatorio Etneo, and installed in the town of Nicolosi, approximately 15 km South of the active vent. The inset plot shows the location of Mt. Etna (yellow square) in Sicily (Italy). The dashed box identifies the summit area. The red dashed arrow indicates the backazimuth between array CONC and the active vent within the South East Crater area; (**b**) Detail of the summit area and craters at Mt. Etna in June 2021 obtained from an Uncrewed Aerial Vehicle survey of the summit area: Bocca Nuova/Voragine (BN/VOR), North East Crater (NEC) and South East Crater (SEC); c) Configuration of the 6-element infrasound array CONC. All maps were produced with the Python packages Matplotlib v3.8 (https://matplotlib.org/) and GDAL v3.7.0 (https://gdal.org/index.html).

For this study, we used acoustic data recorded by a temporary 6-element infrasound array deployed at Mt. Etna in June 2021. The array was installed and operated by the University of Liverpool and the Istituto Nazionale di Geofisica e Vulcanologia (INGV sezione di Pisa and INGV-Osservatorio Etneo) at the Mt. Conca site (Fig. 2a, CONC), approximately 6 km from the active vent within the South East Crater (SEC) area (Fig. 2b). The array had an aperture (i.e., largest distance between any two sensors) of approximately 70 m (Fig. 2c) and was instrumented with IST2018 infrasound microphones^38^ (full-scale range of 480 Pa peak-to-peak, flat response between 0.1 and 40 Hz). Data were recorded on-site with a sampling frequency of 100 Hz and 24-bit resolution.

### Thermal IR

Mt. Etna is routinely monitored by INGV-Osservatorio Etneo (INGV-OE) with an extensive multi-parameter sensor network, including thermal IR cameras. Here, we used thermal imagery of the ENT camera recorded at the site located in Nicolosi (Fig. 2a), approximately 15 km from the active summit craters. The site is equipped with a Flir A40M camera recording IR images in the $7.5 - 13\,\upmu {\text{m}}$ band, with a field of view of 640 × 480 pixels and thermal sensitivity of $80\;{\text{mK}}$ at $25^\circ$^17, 39^. The camera records images with a sampling rate of $0.5\;{\text{Hz}}$, and provides a spatial resolution of 1.3 μrad ($\sim 15\;{\text{m}}$ at the ENT site). We chose this site as it provides the best trade-off between data availability, spatial and temporal resolution, and the ability to track the development of the eruptive plume during paroxysmal activity.

### X-band radar

Volcanic plumes from Etna are also observed using a dual polarimetric X-band radar, managed by the Italian Department of the Civil Protection, part of the national weather radar network^40^. The X-band dual-polarization radar, located at the international airport of Catania (distance of about 32 km from the SEC), has a wavelength of 3.1 cm (9.6 GHz), peak power of 50 kW and half-power beam width of 1.3°. The radar scans a volume defined by an area of 160 × 160 km^2^ and a height of 20 km, recording data along 12 elevation angles every 10 min.

## Results

**Figure 3:**
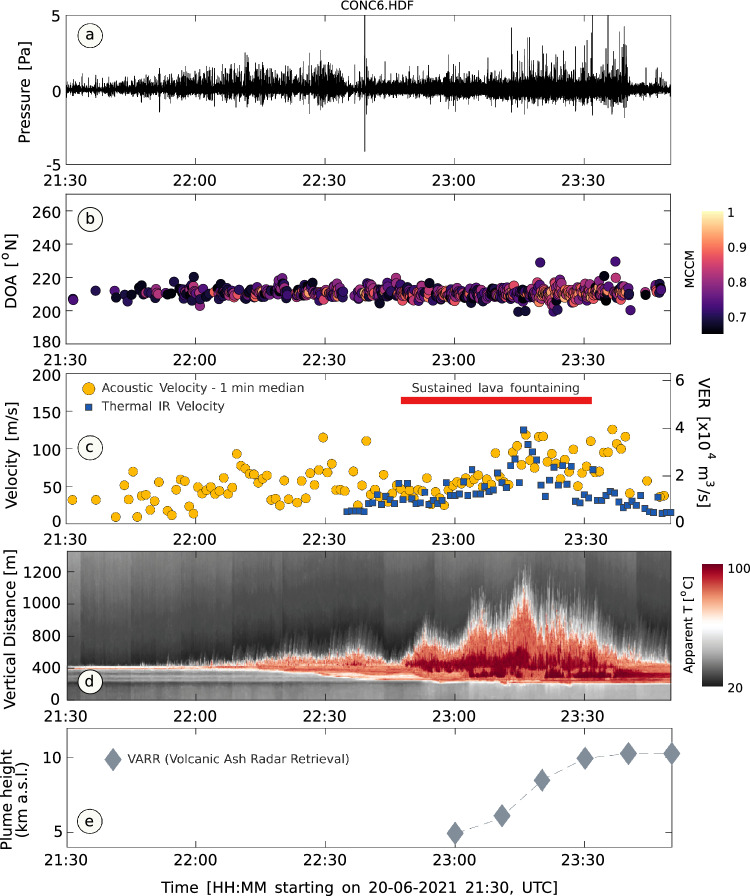
Time series of VER and flow velocity at the vent (infrasound array and thermal IR), and plume height (X-band radar data) during paroxysmal activity on 20 June, 2021. (**a**) Infrasound signal through the paroxysm, recorded by the central sensor of the a temporary 6-element infrasound array at approximately 6 km from the active vent (Fig. 2a, c); (**b**) Time series of high-quality (MCMM > 0.65) Direction of Arrival estimates throughout the paroxysm; (**c**) Time series of flow velocity at the vent estimated from integration of infrasound data (yellow circles) and analysis of thermal IR data (blue squares); (**d**) time series of thermal IR data from the ENT site (Fig. 2a). The plot is produced from stacks across a vertical section (through the crater area) of each time-lapse thermal IR image (one image every 2 s); (**e**) Time evolution of plume height estimated from X-band radar reflectivity data (uncertainty on plume height measurements is +/− 300 m).

Here, we have investigated an episode of paroxysmal activity that occurred on 20 June, 2021 starting at approximately 21:30 (UTC), lasting for about 2 h (Fig. 3a). For this event, INGV-OE issued two Volcano Observatory Notice for Aviation (VONA) with color code Red (the highest level of concern) at 22:01 and 22:19 UTC^41, 42^; INGV-OE reported lava fountaining at the SEC and a plume drifting towards the East-South East. However, the height of the plume could not be estimated in real-time during the paroxysm.

### VER and flow velocity from infrasound array data analyses

We analysed infrasound data recorded at the CONC site (Fig. 2a, c) during paroxysmal activity on 20 June, 2021. Infrasound arrays allow to identify windows of coherent signal and the direction of their source region, providing an effective tool for discrimination of eruptive activity from other signals. We processed the CONC data using slowness inversion^43^ to obtain a time series of Direction of Arrival (DOA), that is the direction from which infrasound energy travels as it crosses the array. The results, in Fig. 3b, show coherent acoustic energy crossing the array from a stable backazimuth in the direction of the active SEC (~ 205°–215° N). We selected data windows corresponding to high quality array locations (MCMM > 0.65) and integrated the pressure signal recorded at the CONC6 microphone (Fig. 2c) to calculate a time series of VER (Fig. 3c). Finally, we used a vent radius of 10 m^44^ to convert VER into flow velocity and calculated its 1-min median, which is shown in Fig. 3c (yellow circles). We also performed calculations for a second paroxysm that occurred on 24 June, 2021 (supplementary material, Fig. S1).

### Flow velocity from thermal IR data analyses

Figure 3c shows a time series of measurements of jet velocity at the vent (blue squares) obtained from analyses of thermal IR data recorded at the ENT site (Fig. 2a) during paroxysmal activity on 20 June, 2021. The measurements, performed with a time step of 1 min, can be compared with the 1-min median jet velocity obtained from acoustic data (Fig. 3c, yellow circles); the two independent time series show excellent agreement throughout the phase of sustained lava fountaining (~ 22:45–23:30). After 23:30, concurrent with waning of lava fountaining, thermal IR estimates of velocity decrease while infrasound-derived jet velocities remain high as acoustic sensors continue detecting individual explosions with no significant associated lava fountaining. Figure 3d shows the thermal IR data from the ENT camera plotted as a time series. The figure, produced by plotting stacks across a vertical section (through the crater area) of each time-lapse thermal IR image (one image every 2 s), allows to track the evolution of the paroxysm in space and time, clearly showing its onset (~ 22:00), peak (22:45–23:40) and waning (Fig. 3c) phases.

### Radar-derived height of the plume and plume model

**Figure 4:**
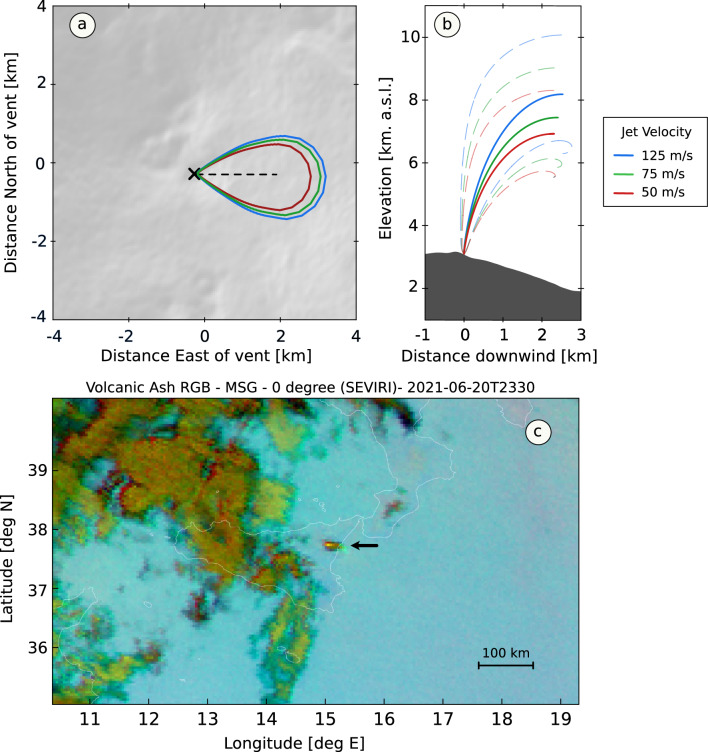
Model and satellite image of the ash plume during paroxysmal activity at Mt. Etna on 20 June, 2023. (**a**) Map view of the modelled plume showing ash drifting towards the East; (**b**) Cross-section (West–East) view of the modelled plume for values of flow velocity of 50, 75 and 125 m/s. Solid lines are represent the height of the plume at its centre and the dashed lines are lower and upper height of the plume as defined by its Gaussian width; (**c**) Composite thermal IR (8.7, 10.8, 12 $\mathrm{\mu m}$ wavelengths) satellite image from the Spinning Enhanced Visible and InfraRed Imager (SEVIRI) at 23:30 on 20 June, 2021 (UTC time). The black arrow indicates the eruptive plume drifting towards the East. Panels (**a**) and (**b**) were generated by the PlumeRise model web interface (https://www.plumerise.bris.ac.uk). Panel (**c**) contains EUMETSAT Meteosat Volcanic Ash RGB—MSG—0 degree product derived from SEVIRI data. SEVIRI data were downloaded from the EUMETSAT data portal (https://view.eumetsat.int/productviewer?v=default). The map was produced with the Python packages Matplotlib v3.8 (https://matplotlib.org/) and GDAL v3.7.0 (https://gdal.org/index.html).

We retrospectively analysed X-band radar data to estimate the temporal evolution of plume height during paroxysmal activity. Figure 3e shows radar detection of a ~ 5 km ash plume starting from 23:00 increasing to a maximum height of 10 km at 23:30. The plume top was estimated at 10 km until ~ 00:00 (21 June, 2021) when it started to progressively wane during the late stages of the paroxysm (supplementary material, Fig. S2). We also used flow velocity estimates from the analysis of infrasound data as input into a 1D model of plume rise^9^. The model output, displayed in Fig. 4 for values of flow velocities of 50, 75 and 125 m/s (VER of $1.6\, \times \,10^{4}$, $2.3\, \times \,10^{4}$, and $3.9\, \times \,10^{4} \,{\text{m}}^{3} {\text{/s}}$ considering a vent radius of $10\,{\text{m}}$) shows the plume drifting towards the East (Fig. 4a), extending vertically to a maximum height of 8–10 km above sea level (Fig. 4b). These results agree with both the radar-derived maximum height (Fig. 3e) and observations of the plume drifting eastwards (Fig. 4c) from satellite thermal imagery collected by the Spinning Enhanced Visible and InfraRed Imager (SEVIRI, 8.7, 10.8, and 12 μm wavelenghts).

## Discussion and concluding remarks

The potential impact of airborne volcanic ash on aviation and infrastructure is well-known. Mitigation of the associated risks depends critically on the ability to issue timely forecasts of atmospheric ash dispersion, and thus, characterize ESP in real- or near real-time. In this study, we have demonstrated an infrasound-based methodology to obtain measurements of VER that holds potential for real-time implementation. We have analysed acoustic data gathered during a paroxysmal event at Mt. Etna on 20 June, 2021, and have benchmarked infrasound-derived measurements of VER (transformed into flow velocity assuming a vent with circular cross-section) with independent estimates of flow velocity at the vent from thermal IR imagery, one of the tools routinely employed by INGV-OE to detect the onset of eruptions, track their evolution and investigate the height of lava fountains during paroxysms.

Flow velocity at the vent and VER are important parameters to constrain eruption dynamics and to evaluate the height of the eruptive plume. Infrasound is a widely adopted tool for volcano monitoring and provides an effective means to measure VER. The main advantages of infrasound over other methods with comparable resolution, such as Doppler radar, are its low installation costs, and simple and rapid data processing that can easily be automated for real-time implementation. Unlike other methods such as those based on analyses of optical or thermal IR imagers, infrasound is not affected by cloud cover. Strong wind can, however, increase noise levels and deteriorate infrasound signal quality although its effects at the recording site can be mitigated through careful sensor installation. Strong directional winds can still severely affect along-path acoustic propagation and obscure signal; installation of multiple arrays at different azimuths with respect to the position of the active vent/s would provide effective mitigation of such adverse wind effects. We also stress that, for application of the methods presented in this study, acoustic arrays are preferred to larger aperture deployments of individual microphones in network configuration owing to their superior capabilities to separate coherent eruption signals from noise. Owing to its characteristics, infrasound is used for operational eruption early warning at Mt. Etna^45^ and worldwide. Implementation of the methodology presented here for VER measurements would be a straightforward addition and a valuable complement to the existing monitoring systems. The main limitation of using infrasound to measure VER is that it requires a well-constrained source mechanism, as well as knowledge of topographic scattering and true signal attenuation, and reflectivity effects at the vent-atmosphere interface. In the case study presented here, as well as other volcanoes^32, 33^, the acoustic source is well-represented by an isotropically radiating monopole (see “Methods” section for details). We adopted the formulation of Lacanna and Ripepe^46^ to estimate VER, which provides a tool to characterize and correct the pressure wavefield for reflectivity at the vent-atmosphere interface in terms of signal frequency and the characteristic source dimension. Their formulation also provides a means to identify non-isotropic radiation patterns in terms of the product of two measurable parameters, that is the characteristic dimension of the source (i.e., the vent radius, a) and the acoustic wavenumber (*k*). The assumption of a pure isotropically radiating source (i.e., an acoustic monopole) holds for values of $ka\le 0.43$; for values of $ka>0.43$ additional corrections accounting for wavefield directivity are required. Finally, correction for signal attenuation from topographic scattering represented by IL is included; IL is evaluated from numerical modelling of acoustic wave propagation (see “Methods” section for additional details).

The acoustic and thermal IR flow velocity time series shown in Fig. 3c show good agreement until approximately 23:15 on 20 June, 2021. After that time the acoustic data continues tracking eruption while the thermal IR signal decreases abruptly. Infrasound waveforms and array DOAs (Fig. 3a, b) confirm that eruptive activity was still ongoing until approximately 23:45; at the same time the X-band radar detected a well-established plume (Fig. 3e). A plausible explanation for the divergence between acoustic and thermal IR data starting from 23:15 is a migration of the fragmentation front deeper into the conduit towards the final stages of paroxysmal activity. In this scenario explosive activity would still be ongoing at depth into the conduit, as detected by infrasound data (Fig. 3a, b), but it would only produce low-level lava fountaining at the surface (which would not be efficiently detected by the ENT camera) while still feeding an airborne ash plume. Infrasound activity declined at ~ 23:45 marking the waning of the paroxysm. A plume between 8 and 10 km persisted until at least 00:30 (supplementary material Fig. S2).

Finally, we have shown that infrasound-derived flow velocities can be used as input into a 1D plume rise model to obtain realistic estimates of the maximum height of the ash column. It should be noted that flow velocities (${v}_{jet}$) were calculated from the infrasound-derived VER and the cross-sectional area of the vent, $S$*,* as ${v}_{jet}=VER/S=VER/\pi$, thus making the conduit radius, $a$, an important parameter. For example, for values of VER of the order of $10^{4} \;{\text{m}}^{3} /{\text{s}}$ , thus similar to those reported in this study, an increase in conduit radius from $10$ to $20\;{\text{m}}$ would result in a decrease in calculated flow velocity of a factor of $\sim 4$. We suggest that a realistic vent radius is used for these calculations, or multiple plume simulations are conducted to assess the sensitivity of plume height to a range of plausible flow velocities at the vent. At Mt. Etna, and many other volcanoes, these issues are increasingly mitigated by the frequent availability of updated digital terrain models. These models, mostly obtained from Uncrewed Aerial Vehicle surveys, allow estimating vent dimensions with very high (< 1 m) spatial resolution e.g.,^44^.

We stress that PlumeRise^9^, the 1D model used in this study, was capable of producing results for a single model in < 1 s, showing its suitability and potential for use in real-time. The results of plume modelling were benchmarked with data from a ground-based X-band radar (Fig. 3e) and the Spinning Enhanced Visible and InfraRed Imager (SEVIRI) satellite (Fig. 4c), which confirmed the maximum height (~ 10 km) and direction (East) of the ash plume, respectively. We note that the one of the objectives of this study was to provide a proof-of-concept for the combined use of infrasound data and plume rise modelling to assess ESP; a full sensitivity analysis of the plume model employed here to its parametrization was beyond the objectives of this work. Plume height is a key ESP as it defines the spreading height of the volcanic cloud in atmospheric ash dispersal models^33^ and it is also often used for assessment of eruption rates. At Mt. Etna plume height is presently derived from analyses of optical images^47^, ground-based radar^40^ and satellite data^48^; successful application of these methods depends on whether specific conditions are met. Methods based on visible cameras are contingent on favorable meteorological conditions (e.g., no cloud cover), can only be used during daytime, and the retrievable height of volcanic plumes is dependent on source-camera distance and the camera field-of-view. Radar data are not affected by meteorological conditions and can be used during both daytime and nighttime; however, detection capabilities depend on source-receiver distance and direction of plume dispersion. Satellite-based measurements of plume height are generally delayed compared to other methods as they require a fully developed (opaque) plume to produce reliable height estimates^47^. Numerical modelling (or alternatively selection of a scenario from an ensemble of pre-computed models) informed by real-time analysis of acoustic data offers a valuable complement to these methods for rapid assessment of plume height.

This study aimed at introducing an alternative methodology for real-time assessment of ESP during eruptions, in particular VER and the top height of the eruptive plume. A number of methods already exist for measurement of ESP; some have briefly been discussed in this manuscript, including advantages and limitations. We have introduced a complementary strategy that relaxes some of the assumptions and simplifications underpinning widely used 0D EPM and overcomes the limitations of other existing techniques. We have demonstrated how analysis of infrasound data allows reliable estimates of VER that can be used to inform 1D plume models, thus, providing real-time estimates of the height of volcanic plumes. These methods would prove especially valuable in the early stages of a volcanic crisis when other measurements are typically not available. At Mt. Etna, for example, rapid information on plume height would be of great importance when issuing warnings to aviation authorities (e.g., VONA), and as a preliminary tool to assess plume dynamics and identify the most likely ash dispersal scenario.

We envision that infrasound-based methods for assessment of ESP, along with numerical modelling, will play an increasingly important role in volcano monitoring operations, and suggest that the implementation of our methodology into an operational workflow will be transformative in influencing decision making and risk mitigation during future volcanic crises.

## Methods

### Thermal IR estimates of jet velocity

Thermal IR images at the ENT site (Fig. 2a) were processed using the algorithm of Mereu et al.^49^; they provide a time series of brightness temperature over the course of a paroxysmal event. The Incandescent Jet Region (IJR), that is the near-vent jet that feeds the volcanic plume, is identified from thermal images as the saturated portion within each frame by evaluating the gradient of the brightness temperature as a function of a given threshold. As a first-order approximation, the height of the IJR is assumed to correspond to the height of the lava fountain^39^, $H_{lf}$, and the jet velocity at the vent ($v_{jet}$) is then calculated as:

$$v_{jet} = \sqrt {2gH}$$

where $g$ is the acceleration of gravity. Detection of the IJR and calculation of $v_{jet}$ are performed for each thermal IR frame and the results averaged every minute (Fig. 3c). The validity of this method depends on considering the IJR a region of non-viscous ballistic flow, a reasonable assumption for volcanic jets near the vent. Additional limitations of the method are that: (1) the correct identification of the IJR can be affected by atmospheric conditions causing variable levels of image saturation; (2) the IJR may be higher or lower than the lava fountain owing to the presence of bombs and lapilli at higher elevation than the lava fountain, or to some of the activity obscured by ash cover^49^.

### Infrasound array processing

Infrasound array data were processed following the methodology of De Angelis et al.^43^. The processing workflow implements least-squares beamforming to evaluate the DOA of coherent energy and the propagation velocity of acoustic waves across the array. The method implements a simple inversion problem:

$${\mathbf{d}} = {\mathbf{Gm}}$$

where ${\mathbf{d}}$ is a data vector of time delay measurements between each pair of sensors within the array, ${\mathbf{m}} = (s_{x} ,s_{y} )^{T} = (sin\theta /v,cos\theta /v)^{T}$ is a model vector of slowness in the East–West and North–South directions, ${\mathbf{G}}$ is a $N \times 2$ matrix of distances between each pair of sensors ($N$ is the number of sensors, $\theta$ is the DOA, $v$ is the acoustic wave velocity across the array). Delay times for each data window analysed are measured using cross-correlation between all pairs of sensors within the array. The results in Fig. 3b were obtained by processing 10-s sliding windows with 50% overlap; a preliminary bandpass filter (0.7–3 Hz, Butterworth, 2-pole) was applied to the data. Only high-quality DOA estimates were selected for plotting, that is those corresponding to a median value of the cross-correlation maximum (MCCM) greater than 0.65.

### Infrasound estimates of VER

We estimated VER by direct integration of the infrasound pressure time series recorded at station CONC6, at central element of the CONC array, (Fig. 2c) following the formulation of Lacanna and Ripepe^46^:

$$VER\left( t \right) = \frac{2\pi r}{{\left( {1 + \left| R \right|} \right)\rho \alpha 10^{IL/20} }}\mathop \smallint \limits_{0}^{t} \Delta P\left( {\tau + \frac{r}{c}} \right)d\tau$$

where $r$ is the source-receiver distance ($5690\,{\text{m}}$), $\rho$ is atmospheric density ($1.1\;{\text{kg/m}}^{3}$), $R$ is reflectance at the vent, $\alpha$ is wavefield directivity, $IL$ is insertion loss, and $\Delta P$ is the recorded pressure time series.

**Figure 5:**
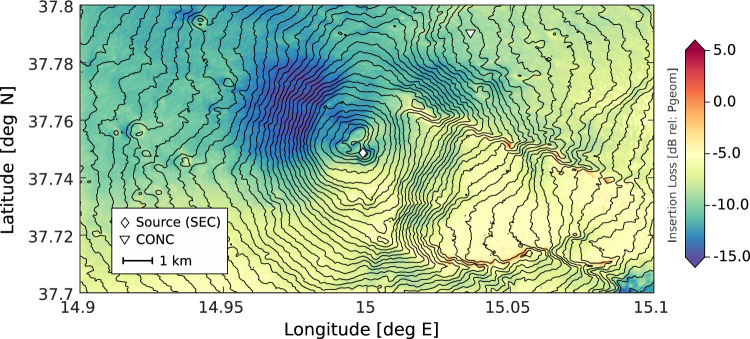
Map of insertion loss at Mt. Etna for a 4.5 Hz source positioned in the SEC area (white diamond). Insertion Loss evaluated from Finite Difference Time Domain numerical modelling of the acoustic wavefield (see “Methods” section of this manuscript). The inverted white triangle shows the position of the CONC array used in this study (IL = − 9.4 dB). Note high signal attenuation (IL ~ − 5 dB) in the area behind BN/VOR and NEC, and the effect of constructive interference from reflections of the acoustic wavefield along the edges of Valle del Bove (IL > 0).

We used values of $R = 0.95$ and $\alpha = 1$ (i.e., isotropic radiation) for a vent radius of $10m$, sound velocity of $330\;{\text{m/s}}$ and a dominant frequency of the acoustic waves of $2\;{\text{Hz}}$^26, 32^. $IL$ represents scattering effect of topography on the wavefield, beyond simple geometric attenuation, which can be calculated from numerical modelling of acoustic wave propagation. $IL$ can be expressed as:

$$IL = 20Log\left( {\frac{{P_{topo} }}{{P_{geo} }}} \right)$$

that is, the ratio between the infrasound pressure calculated at a given position from numerical modelling ($P_{topo}$) and the expected pressure considering only the effect of geometrical spreading ($P_{geo}$). Figure 5 shows a map of IL at Mt. Etna obtained from 3D Finite Difference Time Domain modelling using the software package infraFDTD^28, 29^. InfraFDTD is a 3D linear acoustic wave propagation model over topography with an absorbing boundary (implemented using perfect matched layers) to prevent spurious reflections at the solid interface between the atmosphere and the ground. We note that there are several alternatives to finite difference modelling for acoustic wave propagation over topography, including the family of open source spectral element codes SPECFEM^50^. Similar to Diaz-Moreno et al.^29^ we implement a Blackman-Harris source function with an amplitude of 10000 kg/m^3^ and a cutoff frequency of 4.5 Hz to include the dominant frequency range of the infrasound recorded during the paroxysm (0.7–3 Hz). The topography of Mt. Etna was derived merging a 0.55 m resolution digital elevation model of the SEC area from UAV surveys conducted in March 2021^44^ with a regional 30-m Advanced Spaceborne Thermal Emission and Reflection Radiometer DEM for the rest of the study area^29^; the resulting area was resampled into a grid of 5 × 5 m cells and the acoustic pressure wavefield was propagated over the topography using 20 grid points per wavelength to ensure numerical stability of the procedures^51^. At each grid point we applied Eq. (3) to produce a map of $IL$ for the study area. We calculated a value of $IL = - 9.4\;{\text{dB}}$ at the CONC site. We note that assessment of VER using infrasound requires estimates of IL along a single or a limited number of source-receiver paths. In light of this, 3D modelling could be replaced with 2D modeling, which would greatly increase computational efficiency (and only requires readily accessible computational resources) at the comparatively minor cost of neglecting second-order out-of-plane effects on wavefield propagation. One additional key consideration on IL mapping, is that significant topographic changes, such as major edifice collapses, would require updating IL maps.

### Plume model and atmospheric data

We used the PlumeRise model of Woodhouse et al.^9^ to estimate the maximum height reached by the volcanic plume. The model is a 3-dimensional description of the rise of volcanic ash columns based on the fluid dynamics of turbulent buoyant plumes in a windy atmosphere. This is achieved by combining an integral model of pure plumes in a horizontal wind field^52^ with an integral model of volcanic columns in a static atmosphere^53^. PlumeRise also accounts for the thermodynamics of heat transfer between hot pyroclasts and the surrounding atmosphere and the effects of a variable atmosphere on the rise of volcanic plumes. Additional details and the full model formulation can be found in Ref.^9^. In this study, we used a model of the atmosphere obtained from the Reanalysis v5 (ERA5) dataset. ERA5, produced by the of the European Centre for Medium-Range Weather Forecasts of the Copernicus Climate Change Service, provides hourly estimates of many atmospheric, land and oceanic climate variables. These data cover the Earth on a 30 km grid and resolve the atmosphere using 137 levels from the surface up to a height of 80 km^54^; we used data corresponding to the grid node that is closest to Mt. Etna, at 23:00 on 20 June, 2021. Values for all additional parameters required by the model are shown in the supplementary material (Table S1) and are based on previous work on plume modelling at Mt. Etna^47^.

### Plume height measurements with X-band radar

We applied the VARR (Volcanic Ash Radar Retrieval) algorithm to the measured co-polar radar reflectivity Zhh (dBZ) to obtain information on the nature and height of the volcanic plume^7, 55^. The algorithm performs tephra classification using a maximum a-posteriori probability criterion and estimates tephra features such as its mass concentration and number-weighted mean diameter. The top height of the plume (HTP) is estimated using a threshold algorithm on the measured Zhh of the probed plume and on the retrieved Ct. This estimate equals the central point of the range bin volume. The distance between the radar and the summit craters and the half-power beam width of the radar are used to reconstruct the radar beam cone; an uncertainty on HTP of ± 300 m is evaluated as the half-aperture (radius) with respect to the axis of the truncated cone in the proximal area above the crater^56^.

### Supplementary Information

Supplementary Information.

## Data Availability

The infrasound data and related metadata used in this study are available at https://zenodo.org/record/8207343. The software used for infrasound array processing is hosted at: https://github.com/silvioda/Infrasound-Array-Processing-Matlab. The PlumeRise model is freely available at https://www.plumerise.bris.ac.uk/. The infraFDTD software is available via request to the author, K. Kim (kim84@llnl.gov). Thermal IR (Fig. 3d), radar (Fig. 3e) and high-resolution UAV digital elevation model data (Fig. 2b) are available via reasonable request to the corresponding authors. All other digital elevation model data used in this study are available at: https://tinitaly.pi.ingv.it/. The EUMETSAT Meteosat Volcanic Ash RGB—MSG—0 degree product derived from SEVIRI data is freely available, for research and education use, from the EUMETSAT data portal (https://view.eumetsat.int/productviewer?v=default) after user registration. The EUMETSAT data used in this study were obtained by SDA under EUMETSAT licenses 99999992, 99999993, 99999997, 99999999.
