# Supplementary material for: Assessment of eruption source parameters using infrasound and plume modelling: a case study from the 2021 eruption of Mt. Etna, Italy
Source: Sci Rep. 2023 Nov 13;13:19857. doi: 10.1038/s41598-023-46160-6 (PMC10645732; doi:10.1038/s41598-023-46160-6)
Supplement: Supplementary file 1 — Supplementary Information. [file 41598_2023_46160_MOESM1_ESM.docx]

**Supplementary material for the manuscript:**

**Low-latency assessment of eruption rates using infrasound: a case study from the 2021 eruption of Mt. Etna, Italy**

Authors: S. De Angelis, L.Zuccarello, S. Scollo, L. Mereu

Corresponding author: S. De Angelis, silvioda@liverpool.ac.uk

**Table S1.** Input parameters for the PlumeRise model shown in Fig. 4 in the main text

| *Model Parameter* | *Value* |
| --- | --- |
| Vent Latitude | 37.7475 |
| Vent Longitude | 15.0009 |
| Vent Elevation | 3250 (m) |
| Vent Radius | 10 (m) |
| Gas Mass Fraction | 0.025 |
| Source Temperature | 1400 (K) |
| Pyroclast Density | 1000 kg/m^3^ |
| No-wind entrainment coefficient | 0.09 |
| Wind entrainment coefficient | 0.5 |

**
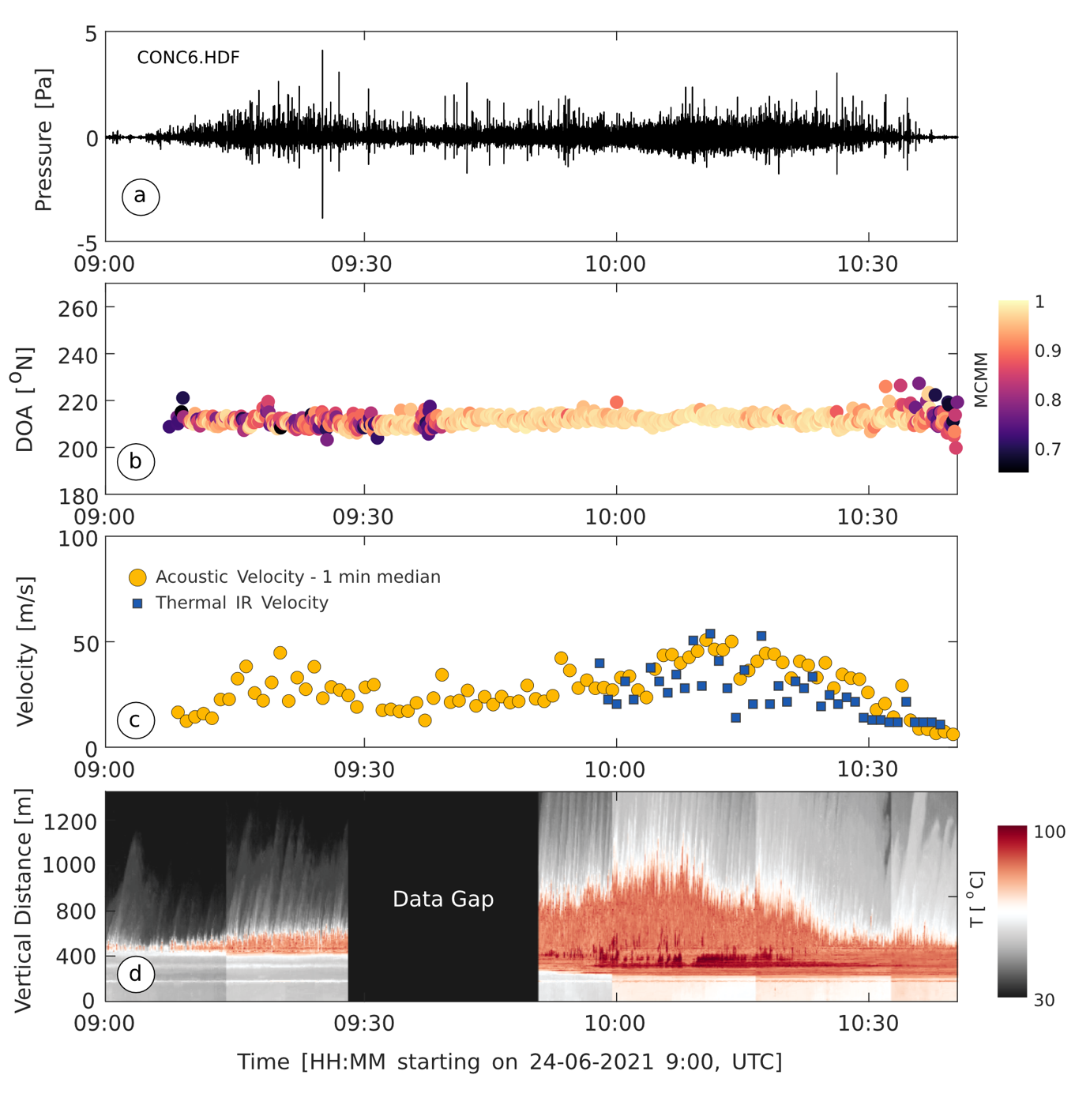
**

**Figure S1.** Time series of VER and flow velocity at the vent (infrasound array and thermal IR), during paroxysmal activity on 24 June, 2021. a) Infrasound signal through the paroxysm, recorded by the central sensor of the a temporary 6-element infrasound array at approximately 6km from the active vent (main manuscript, Fig. 2a, 2c); b) Time series of high-quality (MCMM > 0.65) Direction of Arrival estimates throughout the paroxysm; c) Time series of flow velocity at the vent estimated from integration of infrasound data (yellow circles) and analysis of thermal IR data (blue squares); d) time series of thermal IR data from the ENT site (main manuscript, Fig. 2a). The plot is produced from stacks across a vertical section (through the crater area) of each time-lapse thermal IR image (one image every 2 seconds).

**Figure S2.** Temporal evolution of ash plume during the 20-21 June, 2021 paroxysm estimated from X-band radar reflectivity data (uncertainty on plume height measurements is +/- 300m).
